# The complete chloroplast genome sequence of *Knema elegans* (Myristicaceae)

**DOI:** 10.1080/23802359.2020.1715278

**Published:** 2020-01-20

**Authors:** Chang-Li Mao, Xiao-Qin Li, Feng-Liang Zhang, Tian Yang, Qi Zhao, Yu Wu

**Affiliations:** Yunnan Institute of Tropical Crops, Jinghong, Xishuangbanna, China

**Keywords:** *Knema elegans*, chloroplast genome, Myristicaceae

## Abstract

*Knema elegans* is a member of Myristicaceae. The *K. elegans* chloroplast genome is found to be 155,691 bp in length and has a base composition of A (30.02%), G (19.31%), C (19.89%), and T (30.78%). The genome contained two short inverted repeat (IRa and IRb) regions (48,122 bp) which were separated by a large single-copy (LSC) region (86,883 bp) and a small single-copy (SSC) region (20,686 bp). The chloroplast genome has 85 protein-coding genes, 27 transfer RNA (tRNA) genes, and 8 ribosomal RNA (rRNA) genes. Further, complete chloroplast sequence of *K. elegans* was aligned together with two species of Myristicaceae and five basal angiosperms species for which the complete chloroplast sequence have been reported. This complete chloroplast genome will provide valuable information for the development of DNA markers for future species resource development and phylogenetic analysis of *K. elegans*.

*Knema elegans* belongs to the genus *Knema* of Myristicaceae, is a tall arbor tree, its seed contains 20.8% solids and can be used as industrial oil (Editorial Committee of Chinese Academy of Sciences Flora 1979). So far, it has been analyzed on fatty acid composition (Wu, Mao, Zhang, Yang, et al. 2015) and as the taxonomic group with the other 10 species of Myristicaceae to discuss the taxonomic position of *Horsfieldia pandurifolia* (Wu, Mao, Zhang, Zeng et al. 2015). In this study, we characterized the complete chloroplast genome sequence of *K. elegans* for phylogenetic analysis. The annotated genome sequence has been deposited Genbank under the accession number MK285564.

The fresh leaves of *K. elegans* were collected in 2017 from Lancang River valley, Yunnan, China (100°07.38′E, 21°52.82′N), at the same time, we also took the seeds and brought them back to the base, its seedlings are planted and preserved in Yunnan Institute of Tropical Crops (YITC) and the number of voucher specimen is 20140610. The genome DNA of *K. elegans* was extracted using the DNeasy Plant Mini Kit (QIAGEN, Valencia, CA), and its remaining DNA was stored in an ultra-low temperature freezer. Genome sequencing was performed using Roche/454, sequencing libraries were prepared by the GS Titanium library preparation kit. The chloroplast genome were assembled using CLC Genomic Workbench v3.6 (http://www.clcbio.com). The genes in the chloroplast genome were predicted using the DOGMA program (Wyman et al. 2004).

The circular genome is 155,691 bp in size, and comprises a large single-copy (LSC) region (86,883 bp), a small single-copy (SSC) region (20,686 bp), and two short inverted repeat (IRa and IRb) regions (48,122 bp). The base composition of the circular chloroplast genome is A (30.02%), G (19.31%), C (19.89%), and T (30.78%). The GC content of whole *K. elegans* chloroplast genome was 39.19%. The chloroplast genome has 85 protein-coding genes, 27 transfer RNA (tRNA) genes, and 8 ribosomal RNA (rRNA) genes. There were 50 genes duplicated in the IR regions. The LSC region contained 64 genes, which including 43 protein-coding genes, 18 tRNA genes and 2 rRNA genes, whereas 6 protein-coding genes and 2 tRNA genes were included in the SSC region. The introns were detected in 10 genes including *rpoB, psbB, atpH, rpl23, rps19-fragment, trnQ-UUG, trnS-GGA, trnV-GAC, ndhH, trnL-CAA* and they all have 1 intron.

To study *K. elegans* phylogenetic relationship with the angiosperms, *Horsfieldia pandurifolia* and *Myristica yunnanensis* of Myristicaceae (Changli, Fenglian, Xiaoqin, et al. 2019; Changli, Fenglian, Tian, et al. 2019) and other complete chloroplast genome sequences of angiosperms were downloaded for analyses. The maximum likelihood phylogenetic was performed using MEGA X (Kumar et al. 2018) (Figure 1). A bootstrap analysis was performed on the resulting phylogenetic tree, and values were obtained after 1000 replications. The result shows that *K. elegans* was clustered with other species and closely to *Horsfieldia pandurifolia* and *Myristica yunnanensis* complete chloroplast genome.

**Figure 1.**
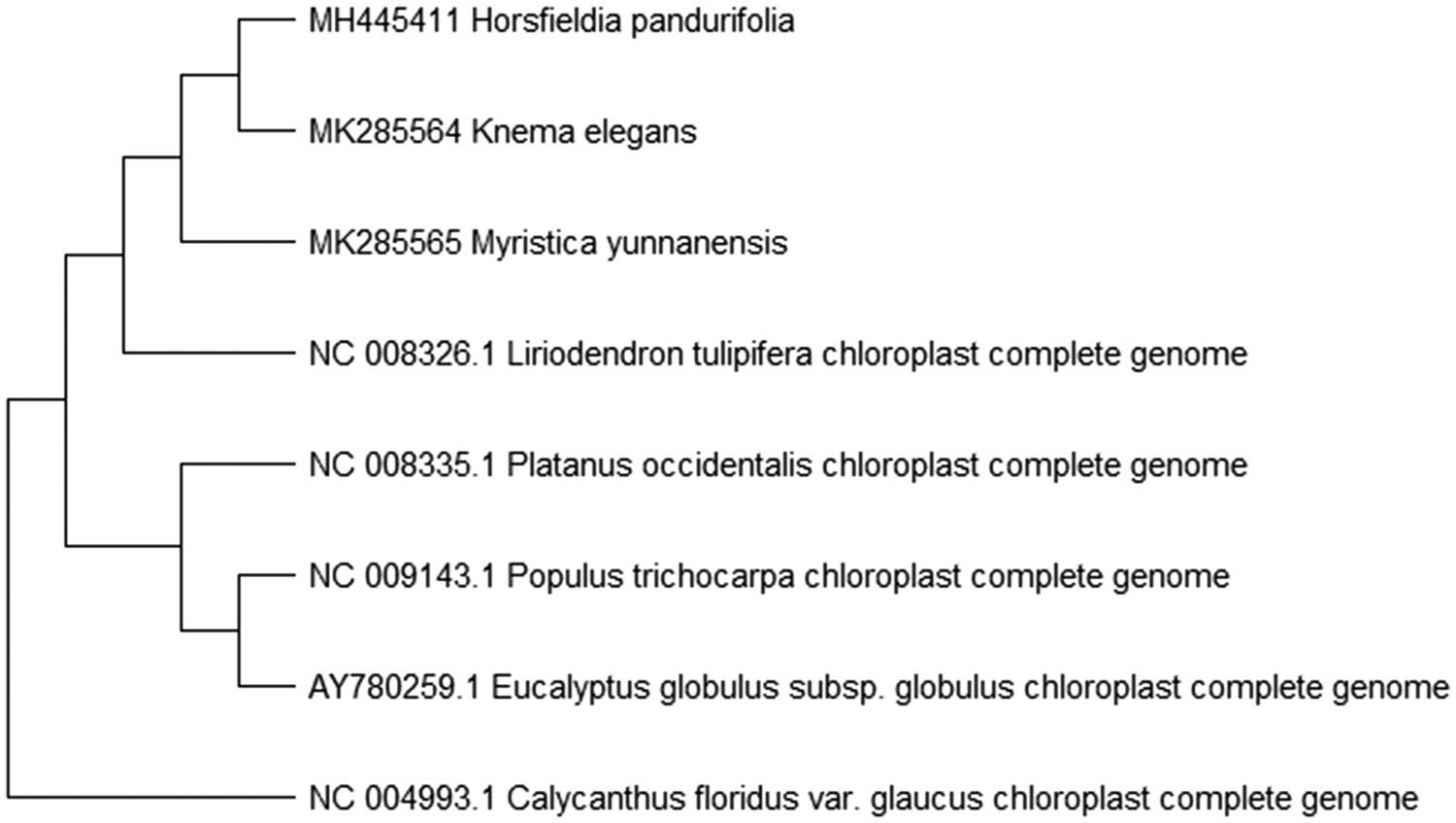
Maximum likelihood phylogenetic tree of *K. elegans* with seven species based on complete chloroplast genome sequences. The gene’s accession number is listed in figure and the data of *H. pandurifolia* and *M. yunnanensis* come from author.

The complete chloroplast genome of *K. elegans* would provide information on the development of molecular markers and phylogenetic analysis in the future.
